# ‘Some anti-malarials are too strong for your body, they will harm you.’ Socio-cultural factors influencing pregnant women’s adherence to anti-malarial treatment in rural Gambia

**DOI:** 10.1186/s12936-016-1255-0

**Published:** 2016-04-11

**Authors:** Fatou Jaiteh, Susan Dierickx, Charlotte Gryseels, Sarah O’Neill, Umberto D’Alessandro, Susana Scott, Julie Balen, Koen Peeters Grietens

**Affiliations:** Department of Geography, The University of Sheffield, Sheffield, UK; Department of Public Health, Institute of Tropical Medicine, Antwerp, Belgium; Medical Research Council Unit, Fajara, The Gambia; Amsterdam Institute of Social Science Research, Amsterdam, The Netherlands; London School of Hygiene and Tropical Medicine, London, UK; School of Health and Related Research, The University of Sheffield, Sheffield, UK; School of Tropical Medicine and Global Health, Nagasaki University, Nagasaki, Japan; Partners for Applied Social Sciences (PASS) International, Tessenderlo, Belgium

**Keywords:** Adherence, Anti-malarials, Pregnancy, Rural Gambia

## Abstract

**Background:**

Despite declining prevalence of malaria in The Gambia, non-adherence to anti-malarial treatment still remains a challenge to control efforts. There is limited evidence on the socio-cultural factors that influence adherence to anti-malarial treatment in pregnancy. This study explored perceptions of malaria in pregnancy and their influence on adherence to anti-malarial treatment in a rural area of The Gambia.

**Methods:**

An exploratory ethnographic study was conducted ancillary to a cluster-randomized trial on scheduled screening and treatment of malaria in pregnancy at village level in the Upper River Region of The Gambia from June to August 2014. Qualitative data were collected through interviewing and participant observation. Analysis was concurrent to data collection and carried out using NVivo 10.

**Results:**

Although women had good bio-medical knowledge of malaria in pregnancy, adherence to anti-malarial treatment was generally perceived to be low. Pregnant women were perceived to discontinue the provided anti-malarial treatment after one or 2 days mainly due to non-recognition of symptoms, perceived ineffectiveness of the anti-malarial treatment, the perceived risks of medication and advice received from mothers-in-law.

**Conclusion:**

Improving women’s knowledge of malaria in pregnancy is not sufficient to assure adherence to anti-malarial treatment. Addressing structural barriers such as unclear health workers’ messages about medication dosage, illness recognition, side effects of the medication and the integration of relatives, especially the mothers-in-law, in community-based programmes are additionally required.

## Background

In malaria endemic countries, pregnant women are particularly vulnerable to malaria infection and its consequences because of physiological hormonal changes, reduced immunity, social vulnerability and difficulty in accessing health care [1–3]. Symptomatic and asymptomatic *Plasmodium falciparum* infections in pregnancy can cause adverse maternal health and poor birth outcomes, with higher risk in primigravidae (first pregnancy) due to a combination of biological and social factors including adolescent’s social position, social acknowledgment process of pregnancy and labor requirements in households [3]. For the control of malaria in pregnancy (MiP) in sub-Saharan Africa, the World Health Organization (WHO) currently recommends individual case management with anti-malarial drugs based on the pregnancy stages, i.e., (i) quinine plus clindamycin in the first trimester and (ii)  artemisinin-based combination therapy (ACT) in the second and third trimester [4]. Additionally, the WHO advises intermittent preventive treatment with sulfadoxine–pyrimethamine (IPTp-SP) during the second and third trimester, given at least 1 month apart, when women attend antenatal clinics [4]. A proposed alternative strategy for MiP, especially in areas where SP resistance is high, is the systematic screening for malaria and consequent treatment of positive pregnant women [5]. For the success of these strategies adherence is crucial. Poor adherence does not only increase the risk of recurrent infections but it may also select resistant parasite strains [6–8].

Research in malaria endemic countries has shown that the decision to adhere to anti-malarials is influenced by, practical reasoning and structural social factors such as, the perceived risks of the medication [3, 9–13] malaria-related beliefs and interpretations of malaria as certain folk illnesses that may delay or stop treatment [12, 14, 15]. Concerns for the safety of the mother and child, for example, when receiving medication late in pregnancy was an often-decisive factor for treatment adherence in Burkina Faso [3], while in Uganda and Malawi bitter medications such as quinine are commonly avoided during pregnancy due to their perceived ability to cause miscarriage and stillbirth [16–18].

In The Gambia, the factors influencing adherence of pregnant women to anti-malarial treatment are largely unknown. Research on MiP to date has focused mainly on the uptake of intermittent preventive treatment with IPTp-SP [16] rather than on the investigation on the use of anti-malarials for case management. This study aimed to explore malaria-related illness perceptions during pregnancy and their influence on adherence to anti-malarial treatment within the socio-cultural context of rural Gambia.

## Methods

### Research design

The study consisted of exploratory ethnographic research ancillary to the cluster randomized trial *“Community Based Scheduled Screening and Treatment of Malaria in Pregnancy for Improved Maternal and Infant Health”* (referred to hereafter as the ‘COSMIC’ study) (Trial Registration: Current Controlled Trials ISRCTN37259296) [22]. The study focused on exploring general perceptions of adherence to treatment for MiP irrespective of diagnosis by RDTs given the low prevalence in the study area at the time of the study. Data were collected through qualitative research techniques, i.e., participant observation, informal conversations and semi-structured interviews. Data were triangulated in order to limit response bias and to form an in-depth understanding of pregnant women’s perceptions related to anti-malarial medication.

### Study site and population

#### Study site

The study was conducted between June and August 2014 in the eastern part of The Gambia (i.e., Upper River Region). The study villages were Demba Kunta Koto, Kuta and Mandinka, selected based on previously collected epidemiological data within the COSMIC study (i.e., malaria incidence, indicators of access to antenatal care (ANC) and uptake of the intervention). Malaria transmission in The Gambia has decreased substantially over the last 10 years [19, 20], revealing increasing heterogeneity, with the eastern part of the country having the highest prevalence [21]. *Plasmodium falciparum* is the main malaria species and transmission in the area is seasonal, occurring primarily between July and December.

#### Study population

The population was mainly *Serahule* and *Mandinka*, and predominantly Muslim. The area is rural with most inhabitants practicing subsistence farming of groundnut and maize and small-scale informal trade. Remittances received from relatives that have migrated to urban parts of the country, elsewhere in Africa, Europe or USA contributed to the livelihoods of some families.

#### Malaria treatment

Anti-malarial treatment in the area was provided at satellite health posts operated by community health nurses (CHNs) and at a major health centre situated 10 km away in Basse Sante Su (a regional level health centre). National guidelines for uncomplicated MiP is oral quinine, but artemisinin-based combination therapy (artemether-lumefantrine) may be used in the second and third trimesters. As this study was ancillary to the COSMIC study [22], in the intervention villages, village health workers (VHWs) followed a training programme in which the burden of MiP and its consequences, and the need for pregnant women to take IPTp-SP as early as possible in the second trimester were highlighted. VHWs were trained to use rapid diagnostic test (RDTs) and to treat positive cases with artemether-lumefantrine.

### Data collection

#### Participant observation

Participant observation consisted of participating in everyday activities in the communities and observing ANC services and activities at the heath posts. These observations offered the opportunity for numerous reiterated conversations with community members. This facilitated trust between the researcher and study participants. Additionally, the observations were important to overcome the bias that is often inherent to self-reporting techniques.

#### Semi-structured interviews

Interviews were recorded and fully transcribed. When not possible and/or inappropriate, the conversation was not recorded but its content was written down in a field diary.

### Sampling

Sampling was theoretical, meaning participants were chosen purposively based on emerging results. Participants identified from the health posts and home visits were selected on criteria such as gender, age, parity, and social position. Furthermore, “snowball” sampling—sampling using participants to identify additional respondents—was utilized to facilitate participant’s confidence in the researcher and hence reduce response bias. The inclusion of other community members, such as health workers and traditional birth attendants, allowed a more holistic understanding of pregnant women’s adherence and reduced the effects of self-reporting bias when only including accounts of pregnant women (Table 1).

**Table 1:** Overview of collected data

Participants (N = 31)	Interviews (N = 31)	Informal conversations (N = 25)	Participant observation (N = 26)
Community health nurse (n = 2)	1	2	4 (health post)
Husbands (n = 4)	4	4	4 (homes)
Mothers-in-law (n = 5)	5	5	5 (homes)
TBAs (n = 2)	2	2	4 (homes, health post)
Women of reproductive age (n = 15)	15	10	5 (homes, health post)
VHW (n = 4)	4	2	4 (health post)

### Data analysis

Data analysis was an iterative process. All observational findings were noted, compiled and analysed at the end of each day. Whilst still in the field, the researcher translated initial recorded interviews from the local language (*Mandinka* and *Serahuli*) into English. These transcripts and observational notes were sequentially analysed in order to inform the interview guide; participant observation and interviews were then conducted to confirm or refute temporary results until saturation was reached. Data were systemized and analysed with NVivo 10 Qualitative Analysis Software (QSR International Pty Ltd. Cardigan UK). The PASS *Malaria in Pregnancy Treatment model* [12] and the *PASS Health Seeking Behaviour model* [23] were used to guide the analytic process. Quotations are presented in this paper to illustrate the range of perceptions within each theme illustrating the respondent’s perspectives [24].

### Ethical approval

Ethical clearance for this study was obtained from the Department of Geography Research Ethics Committee, The University of Sheffield, Sheffield (UK), the Institutional Review Board of the Institute of Tropical Medicine, Antwerp (Belgium) and the of The Gambia Government/MRCG Joint Ethics Committee. The interviewers followed the Code of Ethics of the American Anthropological Association (AAA). The village leaders (*Alkalos*) and all interviewees were informed before the start of the interview about project goals, the topic and type of questions as well as their right to decline participation, to interrupt or withdraw from the conversation at any time. Oral informed consent was obtained before each interview, which was documented by the researcher. Oral consent was favoured since participants were not at any particular risk and moreover, within the local communities, the act of signing one’s name on a piece of paper was expected to bring about mistrust towards the research team [25, 26] as this is not customary practice. Anonymity and confidentiality were guaranteed by using only descriptive identifiers and assigning a unique code number to each informant.

## Results

### Adherence to anti-malarial treatment during pregnancy

Mothers in general explained that adherence to anti-malarial treatment during pregnancy was problematic and that pregnant women regularly threw away their medication or stored it at home. Adherence to treatment would reportedly stop after 1 or 2 days.

*“There are a lot of such cases in this area where pregnant women throw away the medication or hide it behind their pillows.” (*TBA, Demba Kunda Kuta)

*“You know, for some pregnant women, when they feel better after a day or two of taking the medicine, they stop and just hide the rest underneath their pillows.”* (Pregnant woman, Demba Kunda Koto)

Main reasons for these perceptions, as outlined below, were: (i) misconceptions on knowledge of MiP; (ii) perceptions of anti-malarial treatment; and, (iii) the influence of the therapy management group on adherence.

### Knowledge of malaria in pregnancy

#### Local concepts of uncomplicated malaria

Although often used in daily life, the term ‘malaria’ was considered to be a medical doctors’ expression. Depending on the context and background, the following local terms are used to refer to malaria: *kajay, sainaabo* and *susula kurango* in Mandinka; and *samama ntong yea* in Serahule. While these local translations are not true synonyms of malaria, people often equate malaria to these local concepts of illness that refer to general fevers. In the case of *susula kurango*, the term can be translated as ‘mosquito sickness’, and as such directly refers to the cause of the sickness.

#### Non-specificity of symptoms of pregnancy and of malaria

Adherence to treatment was complicated by the lack of recognition of malaria symptoms and their similarity with symptoms of pregnancy itself. Symptoms of uncomplicated MiP mentioned by women included fever, headache, vomiting, lack of appetite, yellow urine, dizziness, body aches, anaemia and general weakness. These same symptoms were also mentioned as common complaints of pregnancy itself and women found it difficult to separate uncomplicated malaria from other pregnancy related illnesses and complaints.

#### Prevention of malaria during pregnancy

When asked how to prevent malaria during pregnancy, almost all respondents mentioned the use of bed nets. Further measures to prevent malaria were mainly related to hygiene, e.g., washing hands before eating food, avoiding food that is contaminated by dirt or flies and cleaning the environment. Some women would also mention Fansidar, referring to IPTp-SP. Most women indicated that without treatment the fetus could develop malaria, leading to concerns about possible complications, such as stillbirths and even maternal death.

### Home treatment

Pregnant women sometimes relied on home treatment as a first remedy for mild malaria symptoms. A number of easily accessible local herbs such as *nebadayo, lemonaso* and *kuninding dolo*, were used at home at the onset of symptoms. These herbs were recommended by older women for cleansing the unborn child in the womb and relieving general pregnancy discomforts, which reportedly included *kajay, sainaabo*, one of the ways of referring to malaria. The local herbs were taken in the form of tea and consumed until women experienced relief from their symptoms. Observational data showed that analgesics were readily available at markets and pharmacies and women indicated that it is common to sponge-dip the body in order to reduce body temperature.

Home treatment was common for mild symptoms. Where home treatments failed, pregnant women would start seeking bio-medical treatment. Additional reasons for home treatment were the time required to look for financial means to attend the health facility; and avoiding the exposure of their pregnancy when women considered this not to be appropriate. This was mostly the case for adolescents who would keep their pregnancy hidden from health workers especially during the early stages due to shyness and shame; and additional and undue attention to the pregnancy, prompting many to fear for negative influences (e.g., witchcraft, *jinn*) on the pregnancy outcome.

In addition, older women (35+) kept their pregnancy hidden as going to ANC created a sense of shame if the woman is pregnant at the same time as her daughter or daughter-in-law. This prompts them to delay exposing their pregnancy and consequently not seeking treatment. It was also indicated that experienced women considered themselves knowledgeable about the treatments for pregnancy requiring less visits to health workers.

### Knowledge of bio-medical treatment

Pregnant women looked for biomedical treatment from the VHW, the health post or health facility in addition to the home treatments. Only a small number of women could mention the name of the medication they received for malaria, such as chloroquine, paracetamol, nivaquine, Coartem^®^. Most women described the medication as a combination of red and yellow capsules, white tablets and red tablets. The term *malaria boro* (malaria medicine) was used to refer to all anti-malarials taken during pregnancy. At the local health posts, CHNs provided verbal instructions on the dosage of the medications, in addition to markings on the plastic bags containing the tablets. However, women reported that certain CHNs did not provide any information on the name of the drugs, the purpose of the medication, potential side effects or the need to complete the medication due to insufficient time related to their workload or, it was argued, because of their perceptions that women with prior pregnancy or MiP experience should be knowledgeable about the medication already. Additionally, some women stated not to complete their treatment regimen in the following cases: (i) when the treatment was considered effective and they got ‘cured’ prior to consuming all doses (e.g., usually meaning a renewed appetite for food and the ability to do household chores); or alternatively (ii) when the medication was considered ineffective because symptoms persisted Table 2.

**Table 2:** Perceived effectiveness of treatment

Quotes	Respondent
*I: Why do you think pregnant women don’t complete their medication?* You can see for some once they start taking the medication, they feel better and after that they won’t complete their medication because they believe they are cured	Adult woman, farmer, Serahuli, Demba Kunda Koto
*I: Why do you think pregnant women do not take their medication as recommended?* R: Some of them, if they are sick and they drink in the afternoon and they feel better before the evening dose, they stop taking the medication	Adult man, VHW, Serahuli, Demba Kunda Kuta
*I: What happened when you drank your anti*-*malarial medication?* R: When I took that medication, my symptoms became worse and I was shivering so I stopped taking the medication	Adult woman, farmer, Serahuli, Demba Kunda Koto
*I: Why do you think pregnant women do not take their medication?* R: Some say that it makes them to vomit, some when they take the medication, they cannot do their chores, and they will be sitting in one place	Adult woman, food seller, Mandinka, Demba Kunda Mandika

### Perceived risks of medication

Pregnant women sometimes reported not completing their treatment due to persisting concerns about consuming ‘strong’ medication during pregnancy. Strong medication was perceived to result in large-sized babies and could therefore lead to risky and painful deliveries. Consuming “strong” and/or “western” medication would also make pregnant women vomit, dizzy and constantly tired, which prevented them from carrying out their household chores. Interestingly, despite the fact that bitter foods in general were widely avoided especially early in the pregnancy as they are perceived to induce miscarriage (e.g., the local herb *jalafato*), the bitterness of biomedical anti-malarials seemed to be less of a problem due to the trust placed upon the “doctor” (i.e., all nurses and doctors working at the health facility) as a provider of “safe medications” (Table 3).

**Table 3:** Perceived risks of medication

Quotes	Respondent
*I: When you were taking the anti*-*malarial, were you fearful that the medication could be harmful to the unborn child?* R: This is possible, if you take some medications and they attack you, and are too strong for your body, you do think to yourself that if I continue taking this medication something unfortunate could happen to me. So it will make you to stop the medication	Adult woman, farmer, Serahuli, Demba Kunda Koto
*I: What other reasons can make a pregnant woman to stop taking her medication?* R: For some people, their unborn babies do not like such medication; therefore they have to stop taking the medication	Adult woman, farmer, Serahuli, Demba Kunda Koto
*I: Do pregnant women normally complain that medication can be harmful to the unborn child?* R: They sometimes complain that the medication can be strong and make the baby to get big. Therefore the delivery process will become harder	Adult man, VHW, Serahuli

### Influence of the therapy management group

Though pregnant women who did not take their anti-malarial treatment were referred to as ignorant and lacking regard for their own health, there was little indication of social pressure to address such behavior. While men were widely seen to be responsible for the welfare of the family, including the health of their wives, they were not knowledgeable about the medication for MiP and were frequently absent from the household during the pregnancy, especially in the Serahuli communities. In most households, the older women, especially mothers-in-law, were influential in health-related decision-making processes. They were considered more knowledgeable about pregnancy-related ailments and local herbs for relieving the symptoms of pregnancy due to their experience in the matter. Informants reported that these influential family members would often deter pregnant women from adhering to anti-malarial treatment, especially when the pregnant woman was not recovering as expected from the symptoms of MiP.

## Discussion

This study documents perceptions on low adherence to anti-malarial treatment despite fairly good bio-medical knowledge of the prevention and complications of MiP. Informants stated that pregnant women would sometimes throw away medication or hide it from health workers. These findings are in line with a previous study on antenatal, birth and postpartum care in which health workers reported difficulties with rural Gambian women’s adherence to bio-medical treatment [27].

For the period of pregnancy, adherence to treatment was difficult due to the non-specificity of the symptoms of pregnancy and malaria. The diffuseness of symptoms made it difficult for pregnant women to distinguish between MiP and general pregnancy ailments. Qualitative studies from settings with heterogenous transmission dynamics have previously highlighted women experiencing difficulties in recognizing MiP [17, 28]. In addition, when attending the health post or receiving treatment from the VHWs, a lack of adequate information provided on the bio-medical treatment was stated, by women, to be problematic. Previous findings on the health sector in rural Gambia also noted tendencies of women being ill-informed about medication by health workers [29, 30]. With limited information on anti-malarial treatment, especially for adolescent women and older women (35+), who did not expose their pregnancy early and had limited contact with health workers, the use of anti-malarials depended on the perceived effectiveness of the medication. Discontinuation of treatment was commonly stated in cases of fast relief or alleviation of symptoms early during treatment as this was perceived as treatment efficacy. Conversely, and in a seemingly contradictory manner, when symptoms persisted after 1–2 days some women discontinued treatment since the medication was seen as inefficacious.

The perception that the medication was *“too strong”,* potentially resulting in large sized babies that make the delivery process difficult and concerns about the side effects such as dizziness were additional motives for women not adhering to their treatment. An alternative strategy to reinforce women’s adherence could be direct observed therapy (DOTs) as it is the case for IPTp-SP. However such a strategy could be difficult to implement especially since the current recommended case management with ACT from the second trimester requires a 3-day course treatment regime. Addressing such strongly held beliefs regarding malaria medication during health promotion and education at the community level is, therefore, important as they have implications for the effective case management of MiP.

Women’s existing trust in health workers and the health system had a positive influence on the acceptance of the medication during pregnancy. In contrast to other studies in sub-Saharan Africa [17, 31, 32], women generally accepted medication from health workers despite the bitter taste. Brabin et al. [16] describe how in 2007 Gambian women accepted SP to be safe because health workers administered the drugs [16], highlighting women’s trust in MRCG and to some extent the larger health system in the Gambia.

Finally, women’s decision to adhere to their anti-malarial treatment during pregnancy was influenced by the advice given by their mothers-in-law. It is important to understand the gender related dynamics of decision-making, resource allocation and authority within a household as it can have an influence on women’s treatment behaviour. The married women settled patrilocally (at the place of residence of their husband) in big compounds shared with the extended members of their husband’s family. Similar to other societies where men are regarded household heads, the husbands here were described as financial providers that may make decisions on financial issues thereby directly or indirectly influencing women’s health. However they were also described as not being knowledgeable about the necessary treatments for pregnancy related ailments. In contrast, the mothers-in-laws formed a core part of pregnant women’s treatment management group. In their role as managing the welfare of their daughters-in-law, the mothers-in-law gave advice on the recognition, care and treatment of pregnancy related ailments. Due to the significant influence on women’s decision-making, integrating mothers-in-law in health activities targeted at pregnant women could improve adherence to treatment since they hold high social status and positions in within their households.

The main study limitation was the non-inclusion of the views of pregnant women diagnosed with malaria by RDT and taking anti-malarial medication at the time of the study. This was not feasible within the study period due to rapidly declining prevalence of MiP. However, the decision not to rely on the accounts of malaria-infected pregnant women had the advantage of minimizing social desirability bias (i.e., apparent contradictions in adherence to treatment due to the tendency to answer questions in a manner that will be viewed favourably by others).

## Conclusion

Strategies for the control of MiP involving anti-malarial treatment will by definition depend on pregnant women’s completion of medication. Using standard health education and awareness programmes to inform women of their risks of MiP and its associated complications are not necessarily enough to increase adherence. Structural barriers such as the type and level of health worker communication with women on the importance of the completion of treatment, illness recognition and side effects of the medication need to be addressed. Additionally, family members such as mothers-in-law should be included in community-based programmes for MiP due to their significant influence on pregnant women’s health.
